# Two new species of the genus *Cyanopenthe* Nikitsky, 1998 (Coleoptera, Tetratomidae) from southwest China

**DOI:** 10.3897/zookeys.874.34724

**Published:** 2019-09-02

**Authors:** Qiaoqiao Ji, Guodong Ren

**Affiliations:** 1 The Key Laboratory of Zoological Systematics and Application, College of Life Sciences, Hebei University, Baoding, Hebei 071002, China Hebei University Baoding China

**Keywords:** polypore fungus beetles, taxonomy, Xizang, Yunnan

## Abstract

The genus *Cyanopenthe* Nikitsky, 1998 is first recorded from mainland China. Two new species, *C. granulata***sp. nov.** and *C. hirtiscutellara***sp. nov.**, are described and illustrated. This genus is redefined, and an updated key to the known species is presented.

## Introduction

The family Tetratomidae Billberg, 1820 within the superfamily Tenebrionoidea Latreille, 1802 consists of approximately 150 extant species belonging to 13 genera of five subfamilies (Nikitsky 1998, 2004, 2005, 2008, 2016; Pollock 2012; Hsiao et al. 2015; Saitȏ and Konvička 2017) and six fossil species belonging to six genera of two subfamilies (Nikitsky 1977; Alekseev 2014; Soriano et al. 2014; Cai et al. 2016; Yu et al. 2016; Hsiao et al. 2018). Among them, 21 extant species of eight genera in five subfamilies are recorded from China, primarily in the southwest and southeast (Nikitsky 1998, 2004, 2005, 2008, 2016; Hsiao et al. 2015; Yoshitomi and Yamasako 2016).

The genus *Cyanopenthe* Nikitsky, 1998 belongs to the subfamily Penthinae Lacordaire, 1859 and contains only four described species in the world (Champion 1916; Nikitsky 1998; Hsiao et al. 2015). *Penthe metallica* Champion, 1916 was described based on single female without locality data. Another two female specimens were discovered from northern India and Bhutan (Nikitsky 2005; Hsiao et al. 2015). Subsequently, a revision of the family Tetratomidae was contributed by Nikitsky (1998). In this work, a new genus, *Cyanopenthe* Nikitsky, 1998, was established and compared with the genus *Penthe* Newman, 1838; *Penthe metallica* Champion, 1916 was designated as the type species of this new genus, and one new species, *C. thailandcia* Nikitsky, 1998, was described. The latter was similarly based on a single female from northwestern Thailand with only a line drawing habitus of the holotype. A line drawing habitus of the holotype of *C. metallica* (Champion, 1916) was also provided by Nikitsky (1998). In 2005, a detailed key to the *Cyanopenthe* species was given by Nikitsky with corresponding figures that including the ovipositor of the holotype of *C. thailandica* Nikitsky, 1998. In a recent work by Hsiao et al. (2015), two new species, *C. taiwana* Hsiao et al., 2015 and *C. leei* Hsiao et al., 2015, were described based on both sexes with color habitus from Taiwan of China, as well as the female ovipositor and the male genitalia; the female ovipositor of *C. metallica* (Champion, 1916) was also presented for the first time, along with a supplementary description, and a key to all species of the genus was provided.

## Materials and methods

The specimens were examined and dissected under a Nikon SMZ800 microscope. Photographs of adult habitus were taken with a Canon EOS 5D Mark III connected to a Canon MP-E 65 mm macro lens. Photographs of other morphological details were taken using a Leica M205A stereomicroscope. Adobe Photoshop 7.0 software was used in image processing. The aedeagus and ovipositor were detached from the body with insect needles, then glued to separate cards and pinned under the specimens. Specimens examined in this study are deposited in **MHBU** (Museum of Hebei University, Baoding, China) and **IZCAS** (Institute of Zoology, Chinese Academy of Sciences, Beijing, China). A double slash (//) separates data of different labels.

Body length was measured from the anterior margin of the clypeus to elytral apex; the terminology of the ovipositor follows Hsiao et al. (2015); absolute measurements are indicated in millimeters (mm).

## Taxonomy

### Key to species of the genus *Cyanopenthe* Nikitsky, 1998 (modified from Hsiao et al. 2015)

**Table:** 

1	Pronotum sparsely and finely punctured; scutellum black, transverse, apex rounded, with distinct, dark rounded impression in middle	**2**
–	Pronotum densely and coarsely punctured or densely granulate; scutellum yellow, bronzed or reddish bronzed triangular, without impression	**4**
2	Antennomeres III–V somewhat slender, projections of antennomeres VIII–IX longer than that of antennomere X; paraproct of ovipositor shorter, 1.2 times as long as wide	***C. thailandica* Nikitsky, 1998**
–	Antennomeres III–V slightly thicker, projections of antennomeres VIII–IX as long as or shorter than that of antennomere X; paraproct of ovipositor longer, 1.4–1.6 times as long as wide	**3**
3	Elytra and abdomen more rounded; anterolateral margin of pronotum more rounded; lateral margins of parameres of tegmen slightly convergent distally; proctiger of ovipositor slightly wider in ventral view	***C. taiwana* Hsiao et al., 2015**
–	Elytra and abdomen more elongate; anterolateral margin of pronotum less rounded; lateral margins of parameres of tegmen subparallel to slightly divergent distally; proctiger of ovipositor slightly slender in ventral view	***C. leei* Hsiao et al., 2015**
4	Pronotum either densely and coarsely punctured throughout or granulate posteriorly with coarse punctures on anterior half of pronotal disc; antennomere V evidently longer than VI in female (Fig. 20; Hsiao et al. 2015: fig. 7); elytral surface with irregular large punctures; posterior margin of abdominal ventrite V less broadened in female (Fig. 19; Hsiao et al. 2015: fig.17); paraproct of ovipositor 1.4 times as long as wide and lateral margins more straighter (Figs 21–23; Hsiao et al. 2015: fig. 26)	**5**
–	Pronotum densely granulate throughout; antennomere V nearly as long as VI in female (Fig. 4); elytral surface with large punctures nearly in rows; posterior margin of abdominal ventrite V more broadened in female (Fig. 9); paraproct of ovipositor 1.7 times as long as wide and lateral margins weakly curved (Figs 14–16)	***C. granulata* sp. nov.**
5	Dorsal side of body blue-violet; pronotum densely granulate, except coarsely punctured in anterior half of disc; scutellum yellow; lateral margins of paraproct of ovipositor somewhat straighter, proctiger almost as long as gonocoxites, gonostylus with long setae (Figs 21–23)	***C. hirtiscutellara* sp. nov.**
–	Dorsal side of body blue; pronotum densely and coarsely punctured; scutellum bronzed or reddish bronzed; lateral margins of paraproct of ovipositor more sinuate, proctiger longer than gonocoxites, gonostylus without setae (Hsiao et al. 2015: fig. 26)	***C. metallica* (Champion, 1916)**

#### Genus Cyanopenthe

Taxon classificationAnimaliaColeopteraTetratomidae

Nikitsky, 1998

698C7BA23A895168881B384BB23AF006

Cyanopenthe Nikitsky, 1998: 29; 2005: 20; 2008: 63; Hsiao et al. 2015: 579; Yoshitomi and Yamasako 2016: 30.

##### Type species.

*Penthe metallica* Champion, 1916 (by original designation).

##### Diagnosis.

Body black, shining, with dark metallic blue or green-blue, covered with dense and black erect pubescence. Head small, dorsal surface with narrow, longitudinal median depression. Eyes lateral, large and protruding. Antennae long, antennomeres VIII–XI (♂) or VII–XI (♀) strongly broadened into a pectinate club. Pronotum transverse, disc weakly convex, ﬂattened laterally with pair of large impressions near base. Prosternal process strongly broadened posteriorly and somewhat roundly truncate apically, slightly exceeding the posterior margin of prothoracic coxae. Scutellum large, triangular or transverse, covered with dense and decumbent yellow to reddish, bronzed pubescence, with or without dark rounded impression at middle. Elytra broadly oval, much wider than pronotum, disc convex, depressed from middle to humeri along lateral margins. Legs slender and long, underside of metafemora with [or maybe without (not mentioned in the previously described species)] dense yellow hairbrush from base to middle in male, metatarsomere I shorter than the remaining tarsomeres combined.

Aedeagus ensiform, parameres slightly shorter than or as long as phallobase. Distal part of parameres divergent in dorsal and ventral view, curved to ventral side in lateral view.

Ovipositor flattened, paraproct elongated, lateral margins subparallel, straight or weakly curved; proctiger semicircular in dorsal view, tapered posteriorly and more or less curved in ventral view.

##### Distribution.

Bhutan, China (Taiwan, Xizang, Yunnan), India, and Thailand.

#### Cyanopenthe granulata sp. nov.

Taxon classificationAnimaliaColeopteraTetratomidae

E4FE4951C770560FBEB35419BCDA02B7

http://zoobank.org/09F5F8A2-92C8-4DEC-9118-43B6659B6FB5

Figs 1–2 , 3–9 , 10–16 , 17

##### Type material.

**Figures 1, 2.**
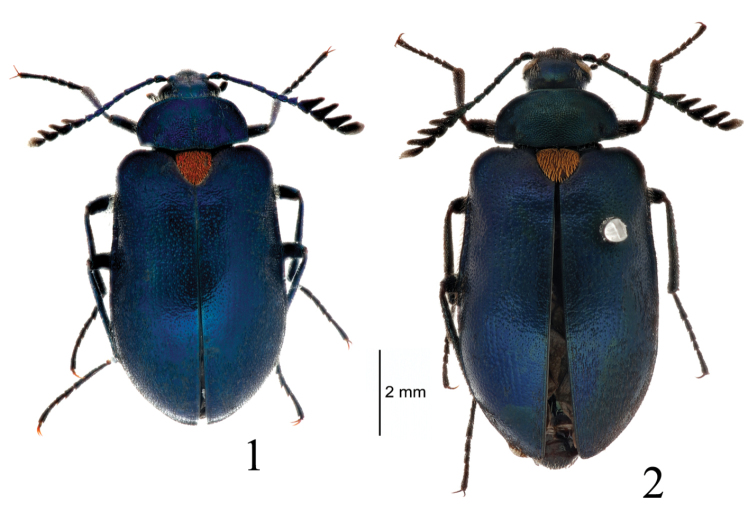
Habitus of *Cyanopenthe granulata* sp. nov. **1** male **2** female.

**Holotype**: ♂ (MHBU) (Fig. 1), with the following labels: “西藏波密县加龙坝村 // 30°02'18"N, 95°15'34"E // 2470 m 2018.VIII.23 魏中华” translated into English as “Jialongba Village, Bomê County, Xizang // 30°02'18"N, 95°15'34"E // Elev. 2413 m, 23.VIII.2018, Zhonghua Wei leg”. Paratype: 1♀ (IZCAS) (Fig. 2), with the following labels:“西藏察隅县上察隅 // 2000 m 杨树桩 // 2005.VIII.24 吴捷” translated into English as “Shang Zayü Town, Zayü, County, Xizang // Elev. 2000 m, Poplar stump // 24.VIII.2005, Jie Wu leg”.

##### Diagnosis.

This species is similar to *C. metallica* (Champion, 1916), but can be distinguished by the following characters (based on females): dorsal side of body green-blue; antennomere V nearly as long as VI; densely granulate on pronotum; scutellum bronzed; elytral surface with large punctures nearly in rows; posterior margin of abdominal ventrite V more broadened; paraproct of ovipositor more elongate (1.7 times as long as wide), lateral margins weakly curved.

##### Description.

Dorsal side of body royal blue, antennae, femora, tibiae and ventral side of body dark blue, some of sternum and abdomen blue-green. Scutellum bronzed, bordered with distinct blue-violet metallic sheen on elytra. Body with dense and black erect pubescence dorsally as well as ventrally. Scutellum with dense and decumbent orange pubescence. Underside of metafemora densely with yellow hairbrush from base to middle in male.

**Figures 3–9.**
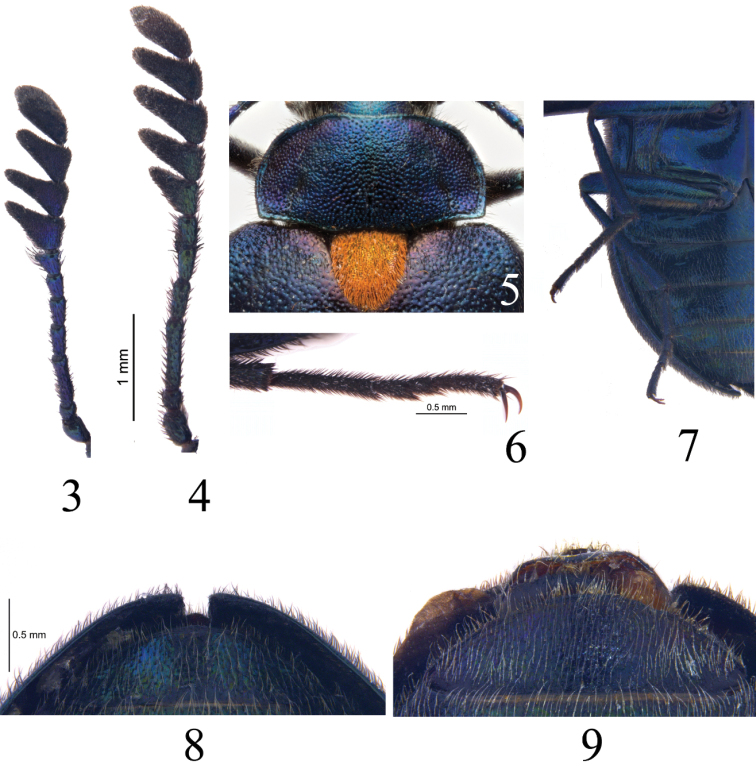
*Cyanopenthe granulata* sp. nov. **3–4** antennae: **3** male **4** female **5** pronotum and scutellum of male **6** metatarsi of male **7** abdomen of male **8–9** abdominal ventrite V: **8** male **9** female.

**Figures 10–16.**
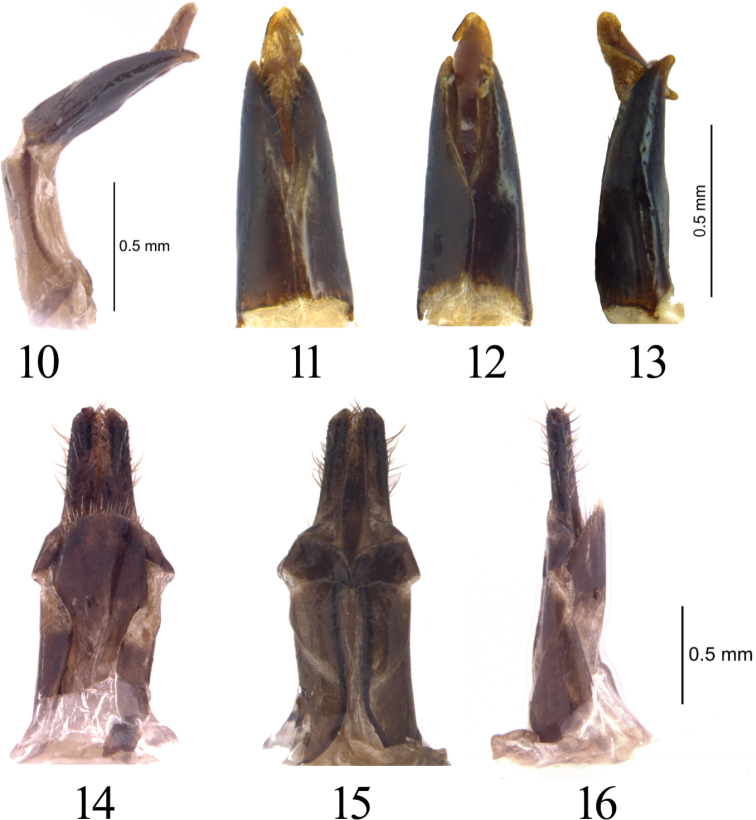
*Cyanopenthe granulata* sp. nov. **10–13** aedeagus: **10** aedeagus lateral view **11–13** parameres dorsal, ventral and lateral view. **14–16** ovipositor dorsal, ventral and lateral view.

**Male** (Figs 1, 3, 5–8, 10–13). *Head* small, length 1.0 mm, width 1.5 mm, densely and finely punctured, dorsal surface with narrowly, longitudinal median depression. Eyes lateral, large and protruding, ratio of eye diameter to interocular space 1.0: 1.9. Maxillary palpomere II elongate-triangular, III suborbiculate, IV obliquely rounded at apex, sides subparallel, surface of extend part somewhat rough and dull, no shining. Antennae (Fig. 3) length 3.8 mm, antennomere I cylindrical, II suborbiculate, III strongly elongate and somewhat clavate, IV–VI clavate, VII somewhat broadened into a pectinate club, approximately as long as projection, VIII–XI strongly broadened into a pectinate club, projections 1.7 times longer than wide; ratio of antennomere lengths as follows: 3.0: 2.0: 6.0: 4.0: 3.2: 2.5: 2.0: 3.2: 3.4: 3.7: 2.8.

*Pronotum* (Fig. 5) transverse, length 1.2 mm, width 2.6 mm, 1.7 times as wide as head. Disc weakly convex, flattened laterally with pair of large impressions extending from base to approximately 1/3 length of pronotum. Surface with dense granules, separated by less than their diameter. Anterior margin slightly sinuate, posterior margin sinuate; lateral margins widest at anterior angles and narrowing posteriorly. Anterior angles rounded, posterior angles rectangular. Prosternal process strongly broadened posteriorly and somewhat roundly truncate apically, slightly exceeding posterior margin of prothoracic coxae. *Scutellum* (Fig. 5) large, triangular, 1.1 times as wide as long; surface densely and finely punctate, without dark rounded impression centrally.

*Elytra* broadly oval, length 6.4 mm, width 4.0 mm, much wider than pronotum. Disc convex, depressed from middle to humeri along lateral margins. Surface with tiny punctures, and large punctures nearly in rows medially on each elytron. Diameter of punctures in spaces between striae 1.7 times smaller than that of punctures in rows.

*Abdomen* (Figs 7–8) oval, linearly narrowed posteriorly, apex rounded. Surface densely and finely punctured. Ventrites with irregular grooves laterally.

*Legs* slender and long. Length of metafemora 2.5 mm, metatibiae 2.1 mm and metatarsi 2.0 mm. Metatarsomere I shorter than II–IV combined. Length ratio of metatarsomeres (Fig. 6) as follows: 10.0: 3.3: 3.0: 8.0.

*Aedeagus* (Figs 10–13) ensiform, parameres as long as phallobase (0.8 mm), phallobase twice as long as wide. Parameres widest at base, lateral margins subparallel, narrowing evenly towards apex, distal part divergent in middle in dorsal and ventral view, curved to ventral side in lateral view. Median lobe 1.2 times as long as tegmen.

**Female** (Figs 2, 4, 9, 14–16). Body larger than male, dark metallic green-blue. Head length 1.1 mm, width 1.6 mm; ratio of eye diameter to interocular space 1.0: 2.3. Antennae (Fig. 4) length 4.1 mm, antennomere VII strongly broadened into a pectinate club, more well-developed than that of male, projection 1.6 times longer than length of antennomere, VIII–X 1.7 times as long as respective antennomeres; length ratio of antennomeres as follows: 3.0: 1.8: 6.5: 3.3: 2.8: 2.6: 3.0: 3.2: 4.0: 3.5: 2.9. Pronotum length 1.4 mm, width 3.0 mm. Elytra length 7.6 mm, width 4.2 mm. Abdominal ventrite V (Fig. 9) protuberant, slightly broadened posteriorly than that of male. Underside of metafemora without yellow hairbrush. Length of metafemora 2.8 mm, metatibiae 2.9 mm and metatarsi 2.4 mm. Length ratio of metatarsomeres as follows: 10.0: 4.0: 2.4: 6.6.

*Ovipositor* (Figs 14–16) flattened, length 1.8 mm, paraproct elongated, 1.7 times as long as wide, lateral margins weakly curved and subparallel; proctiger semicircular in dorsal view, tapered posteriorly in ventral view.

##### Distribution.

China: Xizang.

##### Etymology.

This species is named from the Latin *granulus*, referring to the densely granulose pronotum.

##### Bionomics.

The holotype was found on a dead wood with fungi of Polyporaceae in the forest (Fig. 24). The paratype was found on a stump of poplar.

##### Remarks.

The variation of color in male and female could be caused by fading or differences between male and female individuals; we are not sure. The aedeagus of the holotype and the ovipositor of the paratype are somewhat damaged.

#### Cyanopenthe hirtiscutellara sp. nov.

Taxon classificationAnimaliaColeopteraTetratomidae

2262B1CB9D4E579EAE1225F31DB086F1

http://zoobank.org/F6D0FF83-7ADA-4D6C-BFEB-4C5FA0AF06F2

Figs 17–23

##### Type material.

**Figures 17–23.**
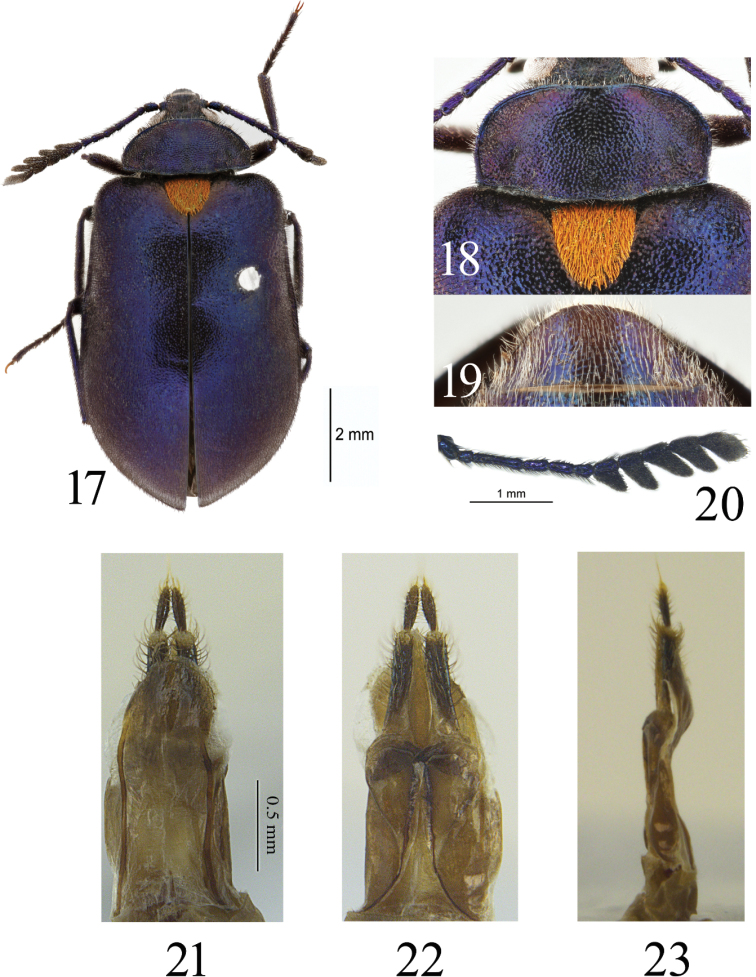
*Cyanopenthe hirtiscutellara* sp. nov. **17** Habitus of *Cyanopenthe hirtiscutellara* sp. nov. **18** pronotum and scutellum **19** abdominal ventrite V **20** antennae **21–23** ovipositor dorsal, ventral and lateral view.

**Holotype**: ♀ (MHBU) (Fig. 17), with the following labels: “2009.VI.2 // 云南独龙江钦郎当 // 1500 m 朱笑愚” translated into English as “2.VI.2009 // Qinlangdang Village, Drungjiang Township, Gongshan County, Yunnan // Elev. 1500 m, Xiaoyu Zhu leg”.

##### Diagnosis.

This species is closely related to *C. granulata* sp. nov. and *C. metallica* (Champion, 1916), but can be distinguished by the following characters (based on female): dorsal side of body blue-violet; pronotum densely granulate, except coarsely punctured in anterior half of disc; scutellum yellow; lateral margins of paraproct of ovipositor nearly straight, proctiger almost as long as gonocoxites, gonostylus with long setae.

##### Description.

Dorsal side of body blue-violet, antennae, femora, tibiae and ventral side of body dark blue, some individuals with sternum and abdomen blue. Scutellum yellow, around scutellum with distinct dark-blue metallic sheen on elytra. Body with dense and black erect pubescence, dorsally and ventrally. Scutellum with dense and decumbent yellow pubescence.

**Female**. *Head* small, length 1.0 mm, width 1.6 mm, densely and finely punctured, dorsal surface with narrowly, longitudinal median depression. Eyes lateral, large and protruding, ratio of eye diameter to interocular space 1.0: 2.0. Maxillary palpomere II elongate-triangular, III suborbiculate, IV obliquely rounded at apex, sides subparallel, surface of extend part somewhat rough and dull, no shining. Antennae (Fig. 20) length 4.0 mm, antennomere I cylindrical, II suborbiculate, III strongly elongate and somewhat clavate, IV–VI clavate; projection of VII about 1.3 times length of the antennomere, VIII 1.7 times longer than width, IX and X 1.6 times longer than width; apices of projections rounded, 1.2 times longer than width; ratio of antennomere lengths as follows: 3.0: 2.0: 7.0: 3.3: 3.3: 2.6: 3.4: 3.3: 3.8: 4.0: 4.4.

*Pronotum* (Fig. 18) transverse, length 1.3 mm, width 2.8 mm, 1.7 times as wide as head. Disc weakly convex, flattened laterally with a pair of large impressions extending from base to approximately 1/3 length of pronotum. Surface densely granulate, except coarsely punctured in anterior half of disc. Anterior margin slightly sinuate, posterior margin sinuate; lateral margins widest at anterior angles and narrowing posteriorly. Anterior angles rounded, posterior angles rectangular. Prosternal process strongly broadened posteriorly and somewhat roundly truncate apically, slightly exceeding posterior margin of prothoracic coxae. *Scutellum* (Fig. 18) large, triangular, 1.1 times as wide as long; surface densely and finely punctured.

*Elytra* broadly oval, length 7.4 mm, width 5.0 mm, much wider than pronotum. Disc convex, depressed from middle to humeri along lateral margins. Surface with tiny punctures and irregular large punctures.

*Abdomen* (Fig. 19) oval, linearly narrowed posteriorly, apex rounded. Surface densely and finely punctured. Ventrites with irregular grooves laterally.

*Legs* slender and long. Length of metafemora 2.7 mm, metatibiae 2.6 mm.

*Ovipositor* (Figs 21–23) flattened, length 1.7 mm, paraproct 1.4 times as long as wide, lateral margins straight; proctiger almost as long as gonocoxites, proctiger semicircular in dorsal view, tapered posteriorly in ventral view; gonostylus with long setae.

**Figure 24.**
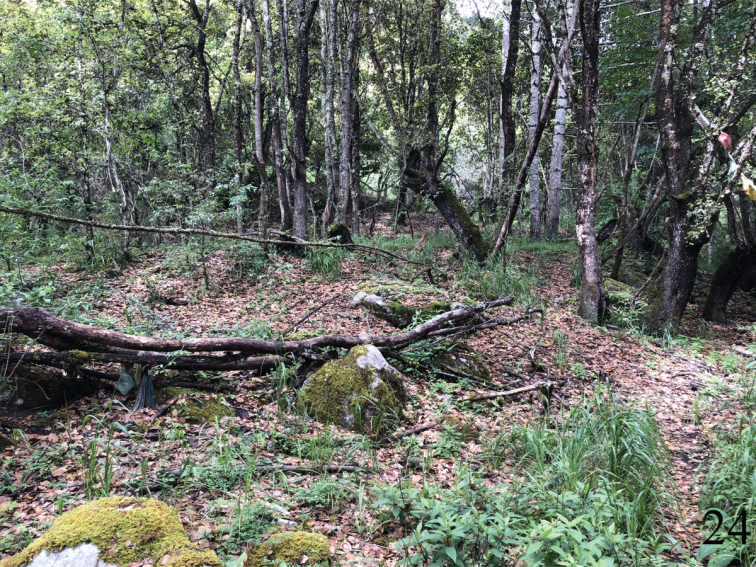
Habitat of *Cyanopenthe hirtiscutellara* sp. nov. Jialongba Village, Bomê County, Xizang.

**Table 1.** Diagnostic characters separating type species and two new species (based on females).

	*C. metallica*	*C. granulata* sp. nov.	*C. hirtiscutellara* sp. nov.
**Color of dorsal side**	Blue	Green-blue	Blue-violet
**Antennomere**	V evidently longer than VI	V nearly as long as VI	V evidently longer than VI
**Pronotum**	Densely and coarsely punctured	Densely granulate	More densely granulate, except coarsely punctured in anterior half of pronotal disc
**Color of scutellum**	Bronzed or reddish bronzed	Bronzed	Yellow
**Punctures of elytral suface**	Irregular	Large punctures nearly in rows	Irregular
**Posterior margin of abdominal ventrite V**	More narrow	More broad	More narrow
**Paraproct**	1.4 times as long as wide	1.7 times as long as wide	1.4 times as long as wide
**Proctiger**	Longer than gonocoxites	Almost as long as gonocoxites	Almost as long as gonocoxites
**Gonostylus**	Without setae	Lost in dissection	With long setae
**Distribution**	Northern India and Bhutan	China (Xizang)	China (Yunnan)

##### Distribution.

China: Yunnan.

##### Etymology.

This species is named from the Latin *hirtus* and *scutella*, in reference to the dense decumbent pubescence on the scutellum.

## Discussion

As far as we know, *Cyanopenthe* species inhabit moist and warm forest habitats, and feed on fungi of Polyporaceae at night in small aggregations or alone; all known species occur in Bhutan, China (Taiwan, Xizang, Yunnan), northern India and northwestern Thailand of Southeast Asia. We believe that more species may be discovered in the Himalayas, Myanmar, Laos, Vietnam and Southern China in the future.

## Supplementary Material

XML Treatment for Cyanopenthe

XML Treatment for Cyanopenthe granulata

XML Treatment for Cyanopenthe hirtiscutellara
